# Measuring the impact of disability on telehealth self-efficacy in five Arab countries: a comparative study in Saudi Arabia and other Arab countries

**DOI:** 10.3389/fpubh.2025.1686216

**Published:** 2025-12-31

**Authors:** Anis Ben Ghorbal, Ibrahim Elsayed Elbatal, A. Aldukeel, Abdelhamid Elshabrawy, Niveen Ibrahim Aly El-Zayat, Samah Zakaria, Heba Ahmed Abd El-Wahab, Dina Mohsen Sabry, Thuraya Elsayed, Suzan Abdel-Rahman, Hatem Semary

**Affiliations:** 1 Department of Mathematics and Statistics, College of Science, Imam Mohammad Ibn Saud Islamic University (IMSIU), Riyadh, Saudi Arabia; 2 King Salman Center for Disability Research, Riyadh, Saudi Arabia; 3 Faculty of Graduate Studies for Statistical Research, Department of Biostatistics and Demography, Cairo University, Cairo, Egypt; 4 Department of Statistics, Faculty of Economics and Political Science, Cairo University, Giza, Egypt; 5 Statistics and Insurance Department, Faculty of Commerce, Zagazig University, Zagazig, Egypt

**Keywords:** Arab countries, disabilities, self-efficacy, telehealth services, technical support, technology adoption

## Abstract

**Introduction:**

This study investigated the factors influencing self-efficacy perceptions of telehealth services, focusing on individuals with disabilities. A comparison was made between Saudi Arabia and four Arab countries: Egypt, Jordan, Libya, and Syria.

**Methods:**

A cross-sectional study design was employed, collecting data through anonymous online questionnaires from a total of 2,065 participants, including 297 individuals with disabilities. Data analysis was conducted using multilevel logistic regression models for the overall sample and a single-level logistic model for disabled individuals. Self-efficacy, the dependent variable, is a binary variable indicating a strong or weak belief in the ability to use telehealth services, while the independent variables include demographic, health-related factors, internet and telehealth usage patterns, and telehealth-related indices.

**Results:**

The most significant variables influencing participants’ perceptions of self-efficacy in using telehealth services were age, expected ease of use, external influence, prior experience with telehealth, availability of technical support, and concerns about technology. In contrast, beliefs about expected obstacles, gender, and chronic disease status were not found to be significant factors.

**Conclusion:**

The study highlights the need to enhance access to telehealth services by improving technological infrastructure, particularly in rural areas. Providing technical support, training, and awareness programs for individuals with disabilities can strengthen their confidence in using these services, especially in countries with lower self-efficacy, such as Libya and Syria.

## Introduction

Telehealth is known for getting healthcare services and information through telecommunications technologies. More formally, the World Health Organization (WHO) defines telehealth as the “delivery of healthcare services, where patients and providers are separated by distance. Telehealth uses Information Communication Technology (ICT) for the exchange of information for the diagnosis and treatment of diseases and injuries, research and evaluation, and for the continuing education of health professionals” (1). Hence, through telehealth utilization, patients can get medical care remotely which makes healthcare access more practical, allowing more effective health management.

Telehealth is considered an essential means for disabled people in particular since eliminating the need for in-person visits ensures more feasible and less burdensome healthcare delivery. As a result, telehealth can significantly reduce any underlying discrepancies in healthcare access. Moreover, minimizing travel requirements to hospitals or clinics helps in reducing cost and time and is particularly beneficial for patients with mobility impairment and multiple disabilities (2).

Despite its numerous benefits, telehealth continues to encounter significant impediments such as technological barriers, privacy concerns, and regulatory and reimbursement issues (3, 4). These barriers are even more challenging for people with disabilities. Disabled people in particular may need more help setting up or using telehealth tools since applications or websites may not be designed for them. For instance, people with vision impairment may face the issue of not being able to access information in telehealth platforms because they may be incompatible with screen readers. In addition, lack of captioning in video conferencing hinders deaf people from interacting virtually with medical professionals. Therefore, it is essential to bridge the digital gap faced by people with disabilities in order to guarantee fair telehealth access and reduce structural inequalities (1).

Based on Bandura’s self-efficacy theory, which emphasizes individuals’ beliefs in their capabilities to organize and execute actions required to manage prospective situations, this study applies the concept to the telehealth context (5). Self-efficacy in telehealth refers to a person’s confidence in their ability to use telehealth technology effectively for healthcare purposes. Hence, individuals with high self-efficacy tend to engage more in telehealth services (such as taking part in virtual consultations) since they feel more capable of using these tools (6). And since there is a considerable reliance on telehealth services among disabled individuals in particular, it is crucial to boost their self-efficacy through comprehensive training, support and ensuring inclusive design of digital health tools (2, 7, 8).

According to Bandura, self-efficacy is shaped by four main sources: mastery experiences, vicarious experiences, verbal persuasion, and physiological or emotional states. In telehealth, these sources translate into users’ previous successful experiences with telehealth systems (mastery experiences), observing or learning from others who effectively use such technologies (vicarious experiences), receiving encouragement and technical support from healthcare providers or peers (verbal persuasion), and overcoming anxiety or discomfort related to technology use (physiological states). Accordingly, the study’s conceptual framework integrates these elements through variables such as prior experience with telehealth, technical support, external influence, and concern about technology to assess how these factors collectively shape individuals’ self-efficacy in using telehealth services (5, 9).

Regarding telehealth development, Egypt, Saudi Arabia, Syria, Libya, and Jordan differ due to variability in healthcare infrastructure, internet access, government support, and political stability. Saudi Arabia is at the forefront of telehealth adoption due to strong government initiatives like Vision 2030 and high infrastructure investment which have enabled the widespread implementation of digital health services (10–12). Hence, Saudi Arabia leads the development of Information and Communication Technology (ICT) among the previously mentioned Arab countries based on its ICT development index score for 2025 (13, 14). This index serves as a tool for tracking and comparing developments in Information and Communication Technology (ICT) across nations and throughout different periods (15). Egypt and Jordan are also making significant progress in telehealth, though they face challenges such as limited rural access and varying levels of digital literacy among their populations literacy among their populations (16–19) In contrast, Syria and Libya lag due to ongoing conflicts, poor infrastructure, and limited resources, which delay the development and accessibility of telehealth services (20–22).

Several statistical models have been employed to study the self-efficacy of telehealth among disabled people, such as logistic regression models (10, 23, 24), structural equation models (7, 25), extended technology acceptance models (26), factor analysis (8, 25, 26), regression analysis (23) and Longitudinal models (23–25).

The present study aims to investigate the factors affecting self-efficacy and the ability to use telehealth services for disabled people in Saudi Arabia compared to other Arab countries. Being a high-income country [according to World Bank (27)], Saudi Arabia is compared with Libya (an upper-middle income country), Egypt and Jordan (examples of lower-middle income countries) and Syria (a low-income country). Comparing Arab countries is highly important in understanding the differences in their self-efficacy in using telemedicine services. This comparison highlights the digital divide between Arab countries in terms of technological infrastructure, internet speed, and adoption of digital transformation. Furthermore, the study addresses a gap in comparative research on telehealth self-efficacy among people with disabilities in Arab countries, incorporating demographic, telehealth and internet usage, and telehealth-related factors within a multilevel analytical framework.

## Methodology

### Study design and study setting

A cross-sectional study was conducted using anonymous online surveys. Data were collected from October 1 to November 20, 2024. The target population included individuals aged 18 years or older from five Arab countries: Saudi Arabia, Egypt, Jordan, Libya, and Syria. The countries were selected based on demographic differences and income levels to examine the impact of economic context on self-efficacy in using telehealth services. Saudi Arabia is classified as a high-income country, Egypt, Jordan, and Libya as middle-income countries, and Syria as a low-income country. This diversity in income classification enables analysis of disparities in digital infrastructure, adoption of digital technologies, and healthcare system models across the surveyed Arab countries. The study was approved by the Ethics Committee under number 00013692. All data were collected anonymously, with no reference to respondents’ personal identities.

### Sample size and sampling technique

The minimum required sample size was calculated to be 384 participants based on a 5% margin of error, *α* = 0.05, and a 95% confidence level for a large population. The actual sample collected exceeded this minimum in all five countries: Saudi Arabia (428), Egypt (402), Jordan (428), Libya (403), and Syria (404). A convenience sampling method was employed in this study. Data were collected through anonymous online surveys distributed via Google Forms and shared on social media platforms including WhatsApp, Telegram, LinkedIn, and Facebook. Inclusion criteria were individuals aged 18 years or older residing in the selected countries, while participants younger than 18 or residing outside the target countries were excluded.

### Questionnaire design

The questionnaire was designed to study the use of telehealth services. It included a set of questions related to participants’ demographic characteristics, such as place of residence, age, gender, educational level, the presence of chronic diseases, and living with a disability that affects daily activities. The questionnaire also included questions about the ease of using the internet and participants’ previous experience with telehealth services.

The questionnaire also aimed to assess participants’ views on the potential benefits of e-medicine services, such as saving time and money (performance expectancy) and ease of use of systems (effort expectancy). Furthermore, the influence of social factors, such as the opinions of friends and doctors, on the use of these services (social influence) was examined, as were available facilities such as technical knowledge and resources (facilitating conditions). The questionnaire also assessed participants’ beliefs about their ability to complete tasks related to using telehealth services (self-efficacy), their sense of security when using the internet to transfer health data (perceived safety), and any barriers they might face, such as the cost and difficulty of technology (perceived barriers). Finally, the questionnaire measured participants’ intention to use these services in the future (intention to use telehealth). The questionnaire is presented in Supplementary File 1.

Table 1 shows Cronbach’s Alpha reliability measure that is commonly used to assess the internal consistency of each set of survey items selected for each telehealth related index including the study outcome (Self-Efficacy). Almost all indices reflect a very high estimate of Cronbach’s Alpha (at least 0.8) reflecting that related items consistently capture the intended construct.

**Table 1:** Cronbach’s alpha reliability measures of all study indicators.

Outcome and telehealth indices	Cronbach’s alpha	No. of items from survey
Self-efficacy (SE)	0.8161	4
Effort expectancy (EE)	0.9204	4
Technical support (TS)	0.8382	4
External influence (EI)	0.8734	3
Expected security (ES)	0.9545	4
Expected benefits (EB)	0.9170	6
Expected obstacles (EO)	0.7855	5
Concern about technology (CT)	0.9621	11
Intention to use (IU)	0.9401	4

### Data and variables

**Figure 1:**
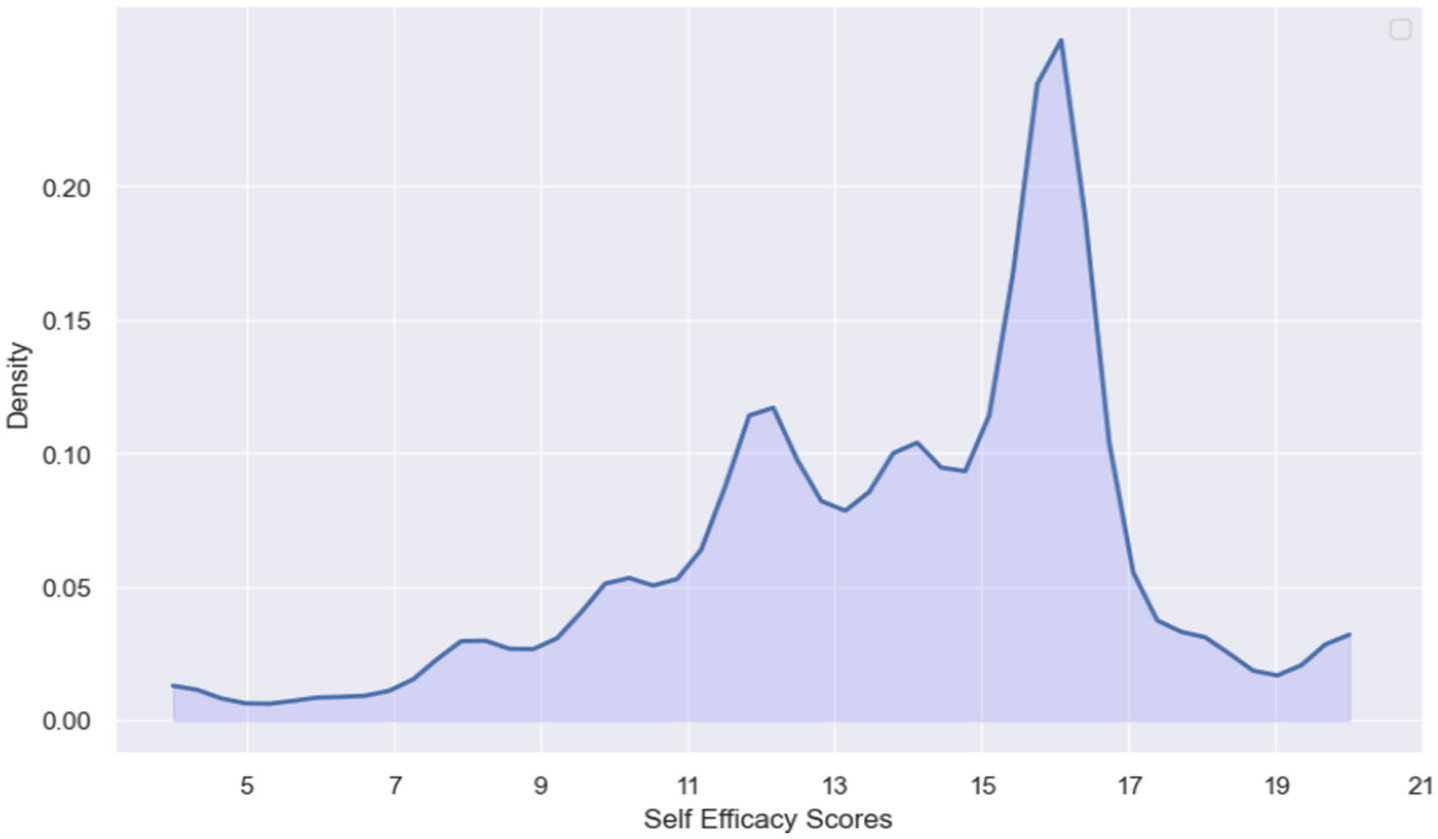
Kernel density plot of self-efficacy scores.

The dependent variable is “Self-efficacy,” which represents a person’s belief in their ability to use the telehealth system. To create this index, the scores of four items on a 5-point Likert scale were summed. Using the Kernel Density plot (see Figure 1), an appropriate cutoff point was chosen to transform the scale of Self-Efficacy into a binary index. Figure 1 shows a bimodal distribution, indicating the existence of two distinct subpopulations. The threshold 15 was chosen to vividly separate these groups. Consequently, the binary index is assigned a value of 0, conforming to the label “weak belief,” if the total score is less than 15, otherwise, it is assigned a value of 1, corresponding to the label “strong belief.”

Sixteen explanatory binary features are thought to influence the target variable. They are classified as follows: (1) *Demographic factors*: sex, age, country, place of residence, and educational level, (2) *Health-related factors*: chronic disease and disability, (3) *Internet and telehealth usage-related factors:* previous experience with using telehealth (Experience) and Ease of net usage (Easy Use) and (4) *Telehealth-related indices*^1^: Technical Support (TS), Expected Obstacles (EO), Intention of Usage (IU) Telehealth services, External Influence (EI), Expected Security (ES), Expected Benefits (EB), Expected Ease (EE), and Concern about Technology (CT).

These independent indicators are measured using the summation of scores of a 5-point Likert scale of some related variables. Technical Support (TS) refers to the facilitating condition of telehealth services. Expected Obstacles (EO) on the other hand reflect the barriers to using telehealth services. Expected Benefits (EB) refer to an individual’s belief in the advantages of using telehealth services. While Expected Security (ES) refers to the degree of security an individual feels when using a telehealth system. Moreover, External Influence (EI) reflects the impact of friends/relatives on an individual’s opinion concerning the usage of telehealth services. Concerns about Technology (CT) represent anxiety about using Technology. In addition, Intention of Usage (IU) demonstrates the willingness to use a telehealth system. And finally, Expected Ease (EE) reflects the degree of ease associated with the usage of telehealth services.

**Figure 2:**
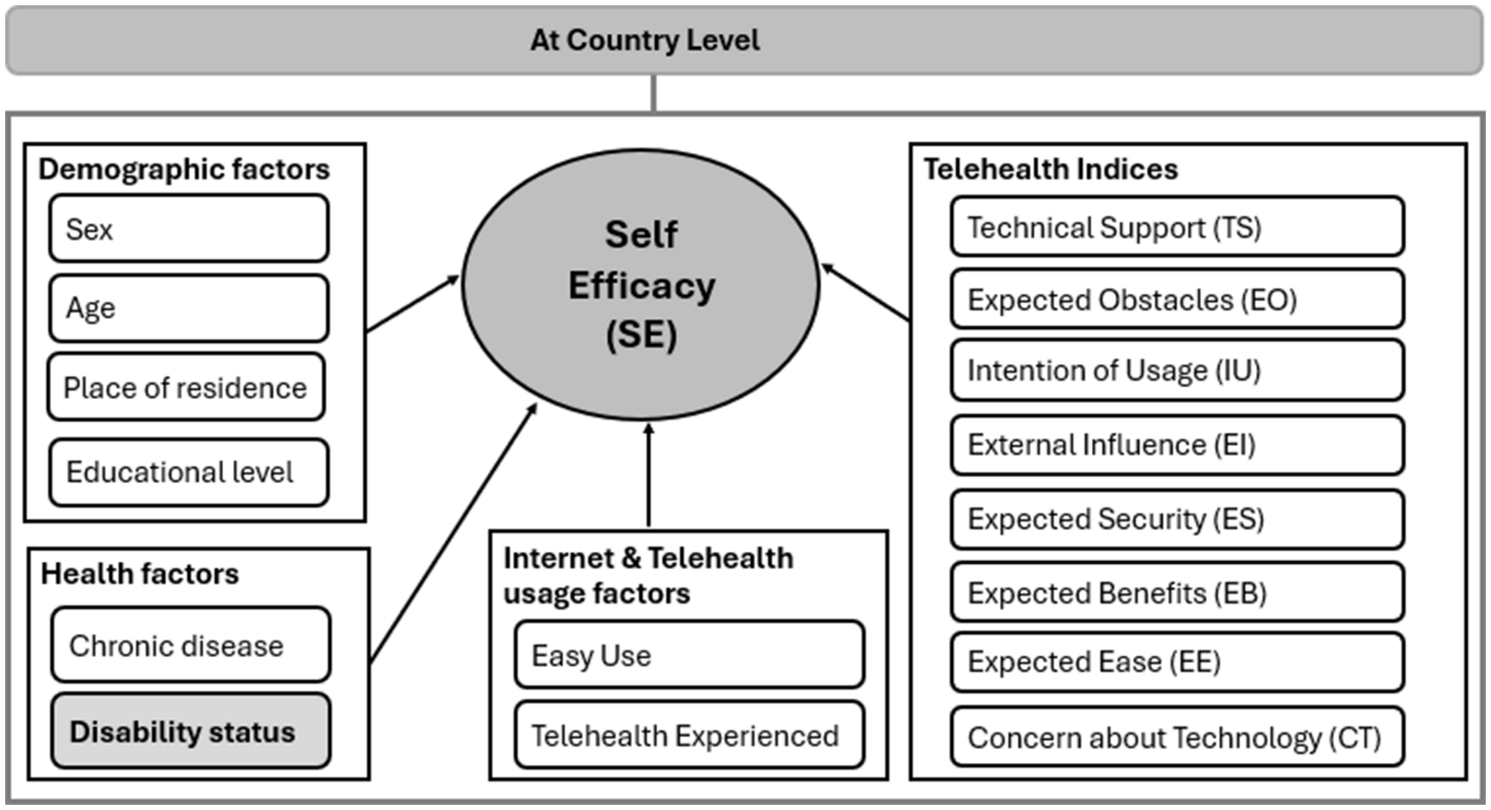
Self-efficacy conceptual framework via two-level modeling approach.

Figure 2 depicts the conceptual framework proposed by the current study to visualize the cause-and-effect relationships between the key independent variables, mainly the telehealth-related indices and the outcome of the study, the person’s belief in their ability to utilize telehealth services, taking into consideration the effect of country-level on the variability of self-efficacy. Figure 2 further involves some other set of control factors: demographic factors, health factors, ease of internet usage, and previous experience with using telehealth. The key purpose of including them is to account for their effect on self-efficacy and hold these factors constant in order to accurately measure the pure effect of the independent factors of interest on self-efficacy.

### Statistical analysis

The current study highlights the importance of multilevel analysis using logistic regression models in examining the determinants of a person’s belief in their ability to utilize telehealth services, primarily when they have a disability. This investigation spans five countries, including Saudi Arabia. The study’s main objective is to investigate the selected factors that affect self-efficacy of using telehealth systems among participants, particularly those with disabilities, in the context of multilevel modeling to consider the impact of distinct levels on the dependent variable (the outcome). The dependent variable is dichotomous, reflecting the person’s perception of his/her self-ability to use telehealth services independently without guidance.

**Figure 3:**
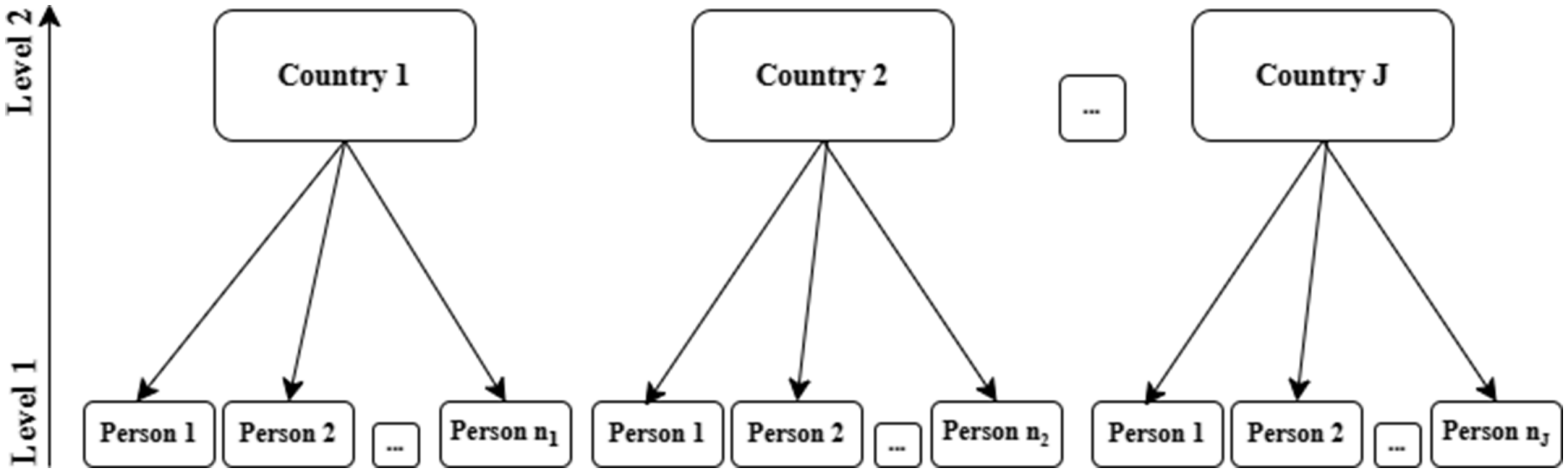
Example of hierarchical data structure (28).

Multilevel logistic regression is employed to analyze the hierarchical data structure where individuals, including those who are disabled, at the lower level (level 1) are nested in countries at the higher level (level 2) (see Figure 3). Such a hierarchical data structure enables one to estimate the odds of binary outcomes as a function of lower level covariates (e.g., person’s age) and higher level covariates (e.g., country’s income), allowing for investigating the effect of group-level variation on the dependent variable (28).

To examine the determinants of participants’ perceptions of their self-efficacy in using telehealth services independently without direct guidance or assistance, multilevel logistic modeling is employed to account for the nuisance of within-cluster homogeneity induced by the hierarchical data structure. Our study begins by fitting three different two-level logistic regression models for different groups of participants based on their disability status. The first two models are applied to the entire sample of participants (*n* = 2,065), while the third model is applied to the subgroup of participants with disabilities (*n* = 297).

The first model (Model 1) is *a random intercept model* that incorporates the fixed effects of 16 individual-specific covariates as well as the country-specific random intercept to model between-country variation in perceptions of ability to use telehealth services. The second model (Model 2) is *the random slope model*. It includes the same covariates as in Model 1. It removes the fixed effect of the individual-specific disability status and incorporates it as a random slope, allowing it to vary across countries. On the other hand, Model 3 is *a random intercept model* is restricted to the disabled group of participants. Furthermore, it incorporates the fixed effect of 15 individual-specific covariates by removing the disability covariate.

Data analysis for this study is carried out using Stata/SE version 17.0 and Python (using Spyder IDE version 5.5.1). The maximum likelihood estimation method is applied to estimate the models’ parameters.

## Results

The current section presents the results of the descriptive analysis conducted. Table 2 represents the distribution of respondents according to their main characteristics. The majority of respondents are females (59%), aged less than 30 years (60%), live in cities (82.5%), and have a university degree or above (66.3%). In addition, most of the respondents do not have previous experience in using telehealth services (85.6%), using the internet is very easy to them (68.7%) and do not have a chronic disease (81%). Unlike other countries, 56% of the participants in Jordan are males, and 56% of the participants in Syria have a high school degree or less. The percentage of respondents who have previous experience in using telehealth services are the highest in Egypt and Syria, followed by Saudi Arabia, compared to the remaining countries.

**Table 2:** Distribution of respondents according to main characteristics by country.

Feature	Country (%)					Overall
	Saudi Arabia	Egypt	Jordan	Libya	Syria	N (%)
Background characteristics						
Sex						
Female	286 (66.8)	245 (60.9)	188 (43.9)	288 (71.5)	209 (51.7)	1,216 (58.9)
Male	142 (33.2)	157 (39.1)	240 (56.1)	115 (28.5)	195 (48.3)	849 (41.1)
Age category						
<30 (young)	329 (76.9)	212 (52.7)	180 (42.1)	281 (69.7)	236 (58.4)	1,238 (60.0)
30–49 (medium)	87 (20.3)	157 (39.1)	175 (40.9)	103 (25.6)	123 (30.4)	645 (31.2)
≥50 (old)	12 (2.8)	33 (8.2)	73 (17.1)	19 (4.7)	45 (11.1)	182 (8.8)
Place of residence						
City	416 (97.2)	315 (78.4)	348 (81.3)	376 (93.3)	249 (61.6)	1704 (82.5)
Rural	12 (2.8)	87 (21.6)	80 (18.7)	27 (6.7)	155 (38.4)	361 (17.5)
Educational level						
High school or less	139 (32.5)	77 (19.2)	97 (22.7)	156 (38.7)	226 (55.9)	695 (33.7)
University and above	289 (67.5)	325 (80.8)	331 (77.3)	247 (61.3)	178 (44.1)	1,370 (66.3)
Health and telehealth/internet-usage related variables						
Disability status						
Disabled	33 (7.7)	57 (14.2)	83 (19.4)	45 (11.2)	79 (19.6)	297 (14.4)
Non-disabled	395 (92.3)	345 (85.8)	345 (80.6)	358 (88.8)	325 (80.4)	1768 (85.6)
Experience						
Experienced	136 (31.8)	168 (41.8)	82 (19.2)	93 (23.1)	168 (41.6)	647 (31.3)
Inexperienced	292 (68.2)	234 (58.2)	346 (80.8)	310 (76.9)	236 (58.4)	1,418 (68.7)
Easy use of internet						
Not easy	34 (7.9)	41 (10.2)	92 (21.5)	53 (13.2)	80 (19.8)	300 (14.5)
Very easy	394 (92.1)	361 (89.8)	336 (78.5)	350 (86.8)	324 (80.2)	1765 (85.5)
Chronic disease status						
Have a chronic disease	46 (10.7)	115 (28.6)	99 (23.1)	61 (15.1)	74 (18.3)	395 (19.1)
Do not have a chronic disease	382 (89.3)	287 (71.4)	329 (76.9)	342 (84.9)	330 (81.7)	1,670 (80.9)
*N*	428	402	428	403	404	2065

Multicollinearity among nominated predictors was checked to ensure model stability and to determine which predictor is well contributing to predict the outcome. All Variance Inflation Factors (VIFs) were below the threshold 5 (ranging from 1.1–2.6), indicating that no severe multicollinearity exists between the independent variables.

Table 3 reports the estimates of the odds ratios and their standard errors of the three models previously presented, as well as the estimates of the variance of the random effects. In addition, the values of the likelihood ratio (LR) test statistics are reported to examine models’ specifications via testing the null hypothesis that the variance between groups at the higher level is not significant (i.e., the single-level logistic model is sufficient to fit the data). The table also reports values of the intraclass correlation (ICC), which represent the proportion of the total variability in the outcome variable that is attributed to the difference between countries. Moreover, models’ accuracy measures using AIC and BIC are reported in Table 3, to evaluate models’ relative quality for fitting the studied sample. Such measures are commonly used to evaluate the model goodness-of-fit against its complexity, consequently, tackling for any potential overfitting.

**Table 3:** Odds ratios and 95% confidence intervals (CIs) of two-level logistic models of self-efficacy, all participants and disabled participants.

Participant groups	All participants				Disabled participants	
	Random intercept (Model 1)		Random slope (Model 2)		Random intercept (Model 3)	
Covariates	OR	95% CI	OR	95% CI	OR	95% CI
Background characteristics						
Male	1.029	(0.820, 1.290)	1.028	(0.819, 1.290)	0.666	(0.310, 1.432)
Age in years	0.975***	(0.963,0.986)	0.974***	(0.963, 0.986)	0.986	(0.957, 1.017)
University+	1.392**	(1.088, 1.782)	1.374*	(1.073, 1.759)	1.302	(0.527, 3.213)
Urban	1.324^•^	(0.970, 1.807)	1.298^•^	(0.952, 1.769)	2.465*	(0.998, 6.089)
Health and telehealth/internet-usage related variables						
Have chronic diseases	0.979	(0.712, 1.346)	0.943	(0.689, 1.293)	1.718	(0.788, 3.748)
Have telehealth experience	1.741***	(1.365, 2.220)	1.723***	(1.350, 2.197)	4.795***	(2.076, 11.08)
Easy use	1.599*	(1.109, 2.305)	1.611*	(1.118, 2.322)	2.042	(0.839, 4.972)
Telehealth-related indices						
TS (agree)	3.840***	(2.769, 5.327)	3.863***	(2.783, 5.361)	6.966***	(2.224, 21.82)
EO (agree)	0.87	(0.690, 1.097)	0.873	(0.693, 1.100)	0.897	(0.411, 1.959)
EB (agree)	1.695**	(1.223, 2.350)	1.688**	(1.217, 2.341)	2.197	(0.732, 6.592)
ES (agree)	1.726***	(1.358, 2.194)	1.726***	(1.358, 2.194)	0.781	(0.351, 1.734)
EI (agree)	1.507***	(1.199, 1.895)	1.507***	(1.198, 1.894)	2.860**	(1.357, 6.027)
CT (agree)	1.472^•^	(0.957, 2.265)	1.481^•^	(0.962, 2.279)	2.123	(0.629, 7.163)
IU (agree)	2.050***	(1.545, 2.719)	2.049***	(1.545, 2.718)	3.670*	(1.353, 9.956)
EE (agree)	2.429***	(1.744, 3.383)	2.483***	(1.781, 3.463)	2.918^•^	(0.950, 8.960)
Disabled	0.832	(0.582, 1.189)	–	–	–	–
_cons	0.035***	(0.016, 0.076)	0.039***	(0.018, 0.082)	0.001***	(0.000, 0.013)
Variance (random effect)	0.201	(0.052, 0.779)	0.359***	(0.358, 0.359)	0.253	(0.022, 2.871)
N	2,065		2,065		297	
No. of groups	5		5		5	
Average no. of obs. Per group	413		413		60	
LR test (chi2 statistic)	44.51***		42.79***		1.81	
ICC	5.77%		9.83%		7.14%	
AIC	2089.6		2092.5		250.1	
BIC	2191.0		2193.8		312.9	

Levels of significance are: ***(<0.001), **(<0.01), *(<0.05), (<0.1), and the initial. ICC is 3.9% with 95% CI (1.1, 13.5%).

The results of Model 1 and Model 2, as displayed, support the use of the multilevel logistic model over the single-level logistic model, given the high values of chi-square test statistics obtained from the likelihood ratio (LR) test (44.5 and 42.8, respectively). Furthermore, both models yield similar results regarding the effects of all incorporated individual-specific covariates on the outcome.

The study attempted to employ different covariance structures between random effects (as in Models 1 and 2). For convergence issues, the “exchangeable” or “compound symmetry” covariance structure was employed in Stata-17 to estimate variance–covariance estimates of the random effects. This covariance structure suggests using equal variances for all random effects and one common pairwise covariance (in the case of Model 2). This specification aims to simplify parametrization and mitigate data complexity, particularly for minor data points at the higher level (29).

Remarkably, Disability status, which is only inserted as a fixed-effect covariate in Model 1, has no significant effect on the dependent variable. Notably, when disability factor has emerged in Model 2 as a random slope, both AIC and BIC have a very slight increase compared to Model 1, yet the ICC of Model 2 increases from 5.8 to 9.8%. That is, adding the participant’s disability status as a random slope explains 69% higher significant variance of perceptions of self-ability in using telehealth services.

Although Model 3 has a high ICC (7%), the chi-square test statistics are insignificant, suggesting that there is no need to fit the data using a multilevel logistic model, while the ordinary binary logistic model is sufficient. Thus, data for the disabled subgroup (*n* = 297) are fitted with the single-level logistic model, referred to as Model 4, to reflect the country-level impact on the dependent variable. Hence, in Model 4, the country-level variable, which has five levels, is represented by four binary variables, with Saudi Arabia serving as the reference category. Results are reported in Table 4, reflecting the highly significant impact of countries on the dependent variable, as recommended by the multilevel logistic models (Model 1 and Model 2).

**Table 4:** Odds ratios and 95% confidence interval limits of single-level logistic model (Model 4) of self-efficacy, disabled individuals.

Covariate	Model 4		
	Odds ratio	Lower 95% CI	Upper 95% CI
**Egypt**	0.241^•^	0.048	1.207
**Jordan**	0.206*	0.048	0.881
**Libya**	0.061***	0.014	0.254
**Syria**	0.054***	0.013	0.219
Male	0.525	0.210	1.314
**Age**	0.944***	0.916	0.974
University+	1.197	0.455	3.149
**Urban**	0.450^•^	0.174	1.162
Have chronic diseases	1.722	0.672	4.412
**Have telehealth experience**	3.484**	1.415	8.579
Easy use	0.734	0.272	1.984
**TS**	14.577***	4.121	51.562
EO	0.796	0.334	1.900
EB	1.542	0.472	5.040
ES	1.220	0.466	3.194
**EI**	2.703*	1.148	6.367
**CT**	3.892*	1.007	15.036
IU	1.003	0.341	2.949
**EE**	4.321*	1.057	17.666

Level of significance are: ***(<0.001), **(<0.01), *(<0.05), (<0.1).

**Figure 4:**
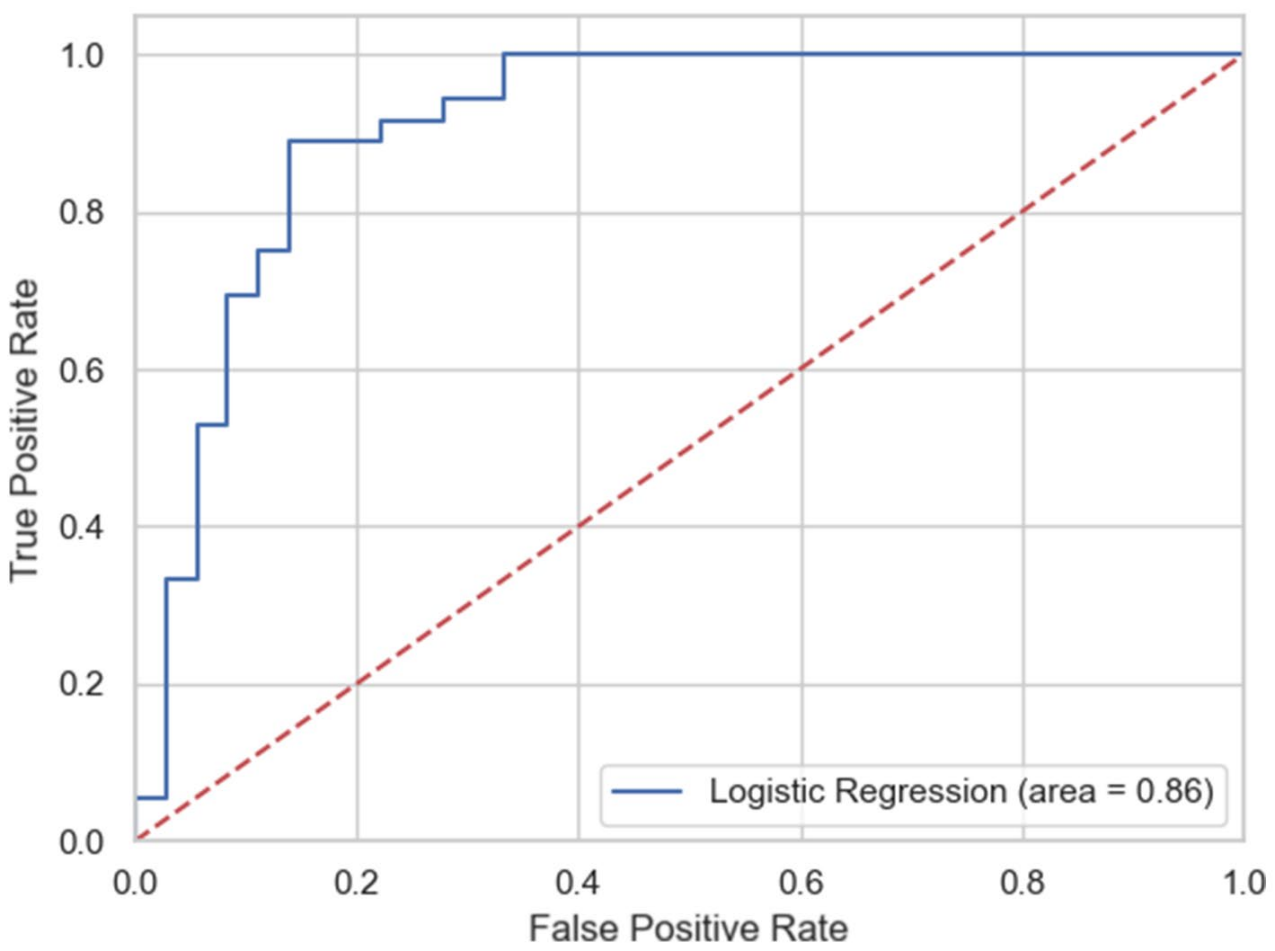
Receiver operating characteristic (ROC) curve of logistic model of self-efficacy, disabled participants.

Moreover, Figure 4 illustrates the Receiver Operating Characteristic (ROC) curve of Model 4, with an Area Under the Curve (AUC) value of 86%, indicating very high predictive power for Model 4. This finding confirms that Model 4 is the most appropriate choice for fitting data that focuses on the disabled subpopulations across different countries, enabling a more efficient and vivid evaluation of their perceptions.

Tables 3, 4 report that across the four models, participants’ gender, chronic disease status, and their beliefs toward the Expected Obstacles (EO) when using telehealth services have no significant effect on Self Efficacy.

Since Model 1 and Model 2 yield nearly identical results, the results of Model 2 (the full model) will be presented for interpretation. Furthermore, Model 4 is more suitable than Model 3 for examining the determinants of Self Efficacy for the disabled group, hence, the results of Model 4 will be investigated.

**Figure 5:**
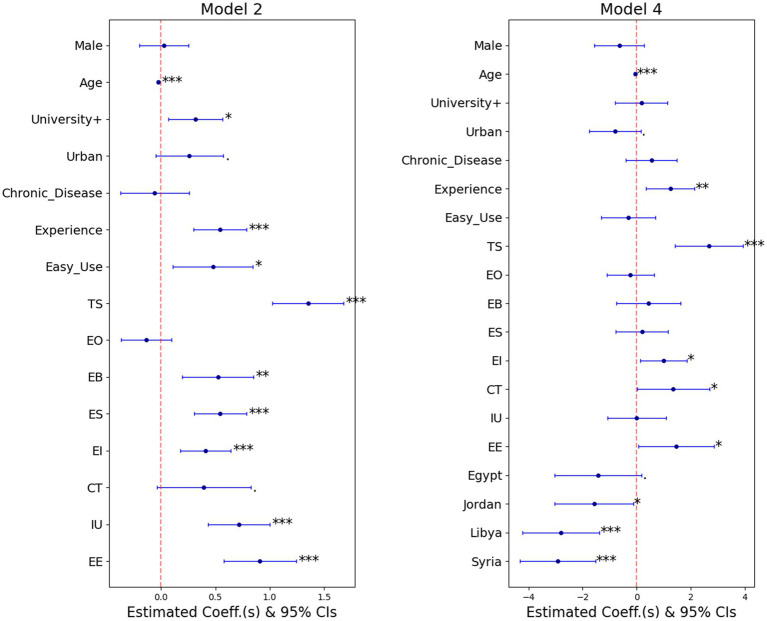
Forest plot of coefficient estimates and 95% confidence intervals of the random slope model (Model 2) and the single-level logistic model for disabled (Model 4) level of significance are: ***(<0.001), **(<0.01), *(<0.05), (<0.1).

To be able to straightforwardly visualize the fixed-effects of the individual-specific covariates across Model 2 and Model 4 by comparing findings from Tables 3, 4, a forest plot is used. Figure 5 depicts the forest plot of the estimated coefficients of all incorporated fixed-effects of the individual-specific covariates as well as their 95% confidence boundaries, where the solid dot indicates the point estimate while the extended connected line defines the 95% confidence limits.

For the whole sample, some covariates show a highly significant impact on the outcome in Model 2. Those covariates are the participants’ positive perceptions of Easiness to Use (Easy Use) telehealth services, participants’ positive beliefs about the Expected Benefits (EB) of telehealth, their Intention to Use telehealth services (IU), and their degree of Expected Security (ES) when using telehealth, as well as their university educational status (University+). They increase the odds ratios of supporting participants’ beliefs in self-efficacy for using telehealth services by 61, 69, 105, 73, and 37%, respectively. Nevertheless, those covariates have no significant impact on the outcome when restricted to the disabled group.

For both Model 2 and Model 4, there are specific covariates that have a significant impact on the outcome. For example, a one-year increase in participant’s age decreases the degree of support for the perception of self-ability to use the telehealth system by 3% (as indicated by Model 2) and 6% (as indicated by Model 4). Additionally, higher support for Expected Ease (EE) of telehealth usage is associated with a 2.5-fold increase in the odds of the outcome (*p* < 0.001), as indicated by Model 2 and a 4.3-fold increase (*p* < 0.05), as indicated by Model 4. Furthermore, higher positive beliefs of External Influence (EI) of friends/relatives on an individual’s opinion concerning the usage of telehealth are associated with increasing the odds of the outcome 1.5 times higher (*p* < 0.001), as indicated by Model 2 and 2.7 times higher (*p* < 0.05), as indicated by Model 4.

Some other covariates have a more significant impact on the outcome among the disabled group than on the entire sample. For example, having any previous Experience with using telehealth systems significantly increases the odds of supporting one’s beliefs about self-efficacy in using telehealth systems by 72% (*p* < 0.001), as indicated by Model 2. At the same time, this amount is 3.5 times higher for experienced than inexperienced (*p* < 0.01), as indicated by Model 4. Additionally, higher positive beliefs in technical support (TS) availability increase the odds by almost four times (*p* < 0.001), as indicated by Model 2, and by nearly 15 times (*p* < 0.001), as indicated by Model 4. Furthermore, any Concern related to anxiety about using Technology (CT) increases the odds by almost 48% (*p* < 0.1), as indicated by Model 2, and by nearly 3.9 times (*p* < 0.05), as indicated by Model 4.

Conversely, participants residing in urban areas are 30% more likely to be confident about their ability to use the telehealth system, as indicated by Model 2, with a significance level of 0.1. In contrast, among the disabled group, participants residing in urban areas are 55% less likely to be confident about their ability to use telehealth services (as indicated by Model 4, with a significance level of 0.1).

Countries have a distinct and significant impact on the outcome. To highlight this impact among the disabled groups, the single-level logistic model is fitted by Model 4. Table 4 shows a decrease in support of self-efficacy in using telehealth systems for all countries compared to Saudi Arabia. This decrease is the highest for disabled participants in both Libya and Syria, as their odds ratios of supporting self-efficacy to use telehealth services are lower than those of disabled participants in Saudi Arabia by 94 and 95%, respectively. Disabled participants in Egypt and Jordan are 76 and 79% less likely than their counterparts in Saudi Arabia to support their self-efficacy in using telehealth systems.

## Discussion

The results showed that telehealth-related indices had a significant impact on self-efficacy for using telehealth services. Technical support, expected benefits, expected security, and expected ease significantly increased individuals’ beliefs about self-efficacy in using telehealth systems. These results are in line with previous studies that have shown that ease of use, lower costs, absence of barriers, and improved communication with providers increase the use of telehealth services and raise their satisfaction (10, 16, 20, 23). Additionally, external influence, concern about technology, and intention of usage played a significant role in shaping self-efficacy, emphasizing the important role of technology in improving usage of telehealth services (21).

In light of Bandura’s self-efficacy model, the impact of telemedicine indicators on confidence in using telehealth services can be further explained. Bandura identifies four main sources of self-efficacy: mastery experiences, vicarious experiences, verbal persuasion, and physiological and emotional states. The results of this study clearly align with these mechanisms. Prior experience with telemedicine represents mastery experience. Previous successful experiences reinforce an individual’s belief in their ability to use the system again, as evidenced by the significantly increased likelihood of self-efficacy among experienced users. External influence reflects vicarious experiences, where an individual’s observation of relatives’ experiences or their encouragement reinforces their belief in their ability to use these services. Technical support embodies verbal persuasion, which reduces hesitation and enhances confidence, and its effect was particularly strong among individuals with disabilities. Finally, concern about technology reflects psychological and emotional states that may lower self-efficacy, as the results showed that increased anxiety is associated with a significant decrease in confidence in an individual’s ability to use telemedicine. These theoretical links confirm that the factors influencing the study align with the psychological foundations of self-efficacy as defined by Bandura’s model (30, 31).

Considering the impact of demographic and health variables, gender had no significant impact. At the same time, age had a negative and highly significant effect, where increasing age is associated with a lower sense of self-efficacy in using these services, which may be attributed to the digital divide or limited access to technology, digital communications required for telehealth services among older age groups. Xie et al. also found that younger individuals were more likely to report using telehealth services than older individuals (24). In contrast, some studies found that older adults are more likely to use telehealth than younger age groups (32), while women were more likely to report using telehealth services than men (24).

Educational level (university or higher) showed a positive and significant effect on using digital health services. This finding is consistent previous studies that showed that educated individuals have digital literacy about healthcare systems and were better able to understand and use the apps (16), as well as being are less likely to hesitate to use telehealth services (21, 24).

The presence of chronic conditions did not significantly impact self-efficacy, in contrast to previous studies that showed that individuals with poorer health or one or more chronic conditions were more likely to use telehealth services, with the likelihood of use increasing with the number of chronic conditions (24). Previous experience with telehealth had a strong significant effect; those with previous experience with the system were more likely to feel self-efficacy. Ease of use showed a significant positive effect, indicating that the user’s perception of the ease of using the system is a key factor in increasing ability to use it. In contrast, individuals who have never used these services may feel hesitant or skeptical about their effectiveness (16).

The non-significant result of the association between gender and self-efficacy in using telehealth services can be explained by the shrinking gender gap in technology utilization and digital literacy across many Arab nations. Due to increased access to smartphones and internet-based services, males and females have equal opportunities for interacting with digital health platforms, thus reducing gender-based disparities in telehealth confidence. Moreover, the non-significance of the chronic disease status might hint that self-efficacy in the use of telehealth is affected more by a person’s prior experience with digital tools, perceived ease of use, and availability of technical support rather than the disease condition of a patient. While people with chronic diseases have more needs than others for telehealth services, this does not translate into a belief that they can use the services without enough familiarity with technology or training. This finding was also reported in the studies of Alboraie et al. (16) and Almathami et al. (10), which indicated that behavioral and technical factors generally take precedence over health status in affecting one’s confidence in telehealth adoption.

The discrepancies between the results of this study and those of previous studies regarding age, gender, and chronic health conditions can be explained by several factors. First, methodological differences between the studies, such as the data collection methods or measurement tools used. Second, the differences in population characteristics between Western and Arab contexts can explain the variation, as customs, culture, and access to technology influence the use of digital health services. Third, the varying quality of telehealth services offered across the studies may alter user experience and trust in the system. Finally, the socioeconomic and political context plays a role in individuals’ access to digital services, which may affect their self-efficacy and willingness to use these services (33, 34).

Among disabled individuals, countries significantly affect self-efficacy in using telehealth systems. Individuals in Jordan had lower beliefs in their ability to use the telehealth services compared to those in Saudi Arabia. Individuals in Libya and Syria, on the other hand, had very weak beliefs in their ability to use the telehealth system. This is consistent with previous studies that have demonstrated that a lack of necessary skills and weak information and communications technology infrastructure are the major challenges limiting the use of telehealth services in Arab countries, including Libya (20).

When comparing the four countries in the study, clear differences were observed in the level of self-efficacy in using telehealth services among individuals. Individuals in Saudi Arabia expressed higher confidence compared to those in Jordan, which may be attributed to the stronger digital infrastructure and higher investments in e-health in Saudi Arabia (35, 36). Conversely, individuals in Libya and Syria showed significantly lower levels of confidence, likely due to weak digital infrastructure and limited access to healthcare services (37, 38). In addition, political stability and broader health coverage in Saudi Arabia may contribute to increased user confidence, whereas the challenges associated with political instability in Libya and Syria reduce individuals’ ability to use telehealth services with confidence (35, 37). These findings underscore the importance of the national context in shaping individuals’ self-efficacy toward health technology.

Among individuals with disabilities, self-efficacy in using telehealth services appears lower in some countries, which may be attributed to several factors. Key among these are difficulties accessing technology and digital services (39, 40), a lack of dedicated technical support (41), and the absence of inclusive digital platforms designed to meet the needs of people with disabilities (42). These challenges can also vary depending on the type of disability; for example, individuals with physical or visual impairments may face different difficulties than those with hearing or intellectual disabilities. This is one of the limitations of the current study, which did not categorize participants by type of disability. These findings highlight the need to improve the digital health technology infrastructure to make it more inclusive and accessible, as well as to provide dedicated technical support for people with disabilities to enhance their confidence and competence in using these services (40, 42).

The multilevel statistical model used provides a deeper understanding of the determinants of self-efficacy in the use of telehealth services. The ICC values indicate that a significant portion of the variance in self-efficacy is attributable to differences between countries, highlighting the importance of the national context. The significance of the random slopes in Model 2 demonstrates that the impact of disability status varies between countries, explaining 69% more of the variance compared to the model relying solely on random intersection. These results indicate that the use of multilevel models is essential to capture the effects of both the individual and national levels, providing a more accurate interpretation of the factors influencing self-efficacy among both the general population and people with disabilities. Furthermore, the predictive power of Model 4 for the disability subgroup (AUC = 86%) confirms that the model captures differences in confidence in using telehealth services, highlighting the importance of considering differences between countries in interpreting self-efficacy outcomes.

The study’s findings highlight the importance of enhancing the self-efficacy of individuals, particularly those with disabilities, in using telehealth services. This can be achieved through improved digital infrastructure, the provision of technical support and training by healthcare institutions, and the design of user-friendly and inclusive systems by technology developers. Communities and organizations of people with disabilities also play a crucial role in raising awareness of the benefits and promoting the use of these services through education and peer support programs, thereby fostering confidence and the ability to use them effectively.

Furthermore, the telehealth experience had a significant positive impact on the self-confidence of individuals with disabilities in using these services. However, it was observed that their confidence in effectively utilizing telehealth tends to decline with age. Gender and place of residence had no significant impact on self-efficacy of using telehealth services among disabled individuals. These findings contradict previous studies that found significant differences in telehealth use based on geographic region, gender, and age where older individuals and women with disabilities were more likely to use telehealth services (32).

The study recommends enhancing access to telehealth services by improving technological infrastructure in rural areas. Technical support and training for individuals with disabilities should be increased to boost their confidence in using these services. Additionally, awareness programs on the benefits of telehealth should be expanded, particularly in countries with low levels of self-confidence, such as Libya and Syria.

A major strength of this study is its large, multi-country sample and focus on individuals with disabilities, a group rarely studied in telehealth research. The use of multilevel analysis allows for a better understanding of both individual and country-level factors affecting self-efficacy in using telehealth services. The study also highlights the importance of telehealth-related indicators such as technical support, safety, and expected benefits in improving confidence and healthcare outcomes.

The study’s weaknesses include not considering some important variables that may affect individuals’ ability to use telehealth services, such as their type of health insurance or their economic status, in addition to the types of disabilities. In addition, the study’s limitations include the potential for sample selection bias due to the use of a convenient sampling method, the reliance on self-reporting which may affect the accuracy of the data, and the exclusion of individuals with insufficient internet access because the study relied solely on online responses.

## Conclusion

This study explored the factors shaping individuals’ perceptions of their self-efficacy in using telehealth services across five Arab countries. The results showed that technical support, safety, and perceived benefits play a crucial role in enhancing individuals’ belief in self-efficacy in using these services. The study also indicated that demographic factors, such as age, educational level, and place of residence, significantly influence how individuals use these services. While the presence of chronic diseases was not significant, previous experience with telehealth and ease of use were key factors that impacted self-efficacy. It also demonstrated that differences between countries significantly impacted individuals’ perceptions of their ability to use telehealth services. The study recommends strengthening technological infrastructure, especially in rural areas, and providing technical support to individuals with disabilities.

## Data Availability

The raw data supporting the conclusions of this article will be made available by the authors, without undue reservation.

## Footnotes

1 Telehealth-related indices are constructed by averaging responses of 5 poits likert scale related items. The scores are then rounded to the nearest integers with range 1–5 and recoded into binary scores, with values at least 4, coded as 1 (“Agree”), otherwise, coded as 0 (“Disagree”).
