# Supplementary material for: Measuring the impact of disability on telehealth self-efficacy in five Arab countries: a comparative study in Saudi Arabia and other Arab countries
Source: Front Public Health. 2025 Dec 31;13:1686216. doi: 10.3389/fpubh.2025.1686216 (PMC12801053; doi:10.3389/fpubh.2025.1686216)
Supplement: Supplementary file 1 [file Data_Sheet_1.docx]

**استبيان عن** **استخدام خدمات الرعاية الصحية عن بُعد**

تُعرف خدمات الرعاية الصحية عن بُعد او الطب الإلكتروني بأنها تقديم الرعاية الصحية من مسافة باستخدام التقنيات الحديثة مثل المواقع الالكترونية وتطبيقات الأجهزة الذكية من قبل المنشآت الصحية أو الممارسين الصحيين للتمكن من التواصل الآمن والمباشر بين المريض والممارس الصحي. وتشمل خدمات الطب الإلكتروني -على سبيل المثال - تقييم التاريخ المرضي، الفحص عن بُعد، التشخيص الطبي للمريض، الاستشارة عن بُعد، متابعة المرضى عن بُعد. يستهدف هذا البحث الأشخاص الذين تتعدى أعمارهم 18 عاماَ. نتائج البحث الحالي قد تتيح الفرصة لوضع خطة توعية حول استخدام الطب الإلكتروني وتحسين الخدمات الطبية المقدمة عن طريق الطب الإلكتروني. مشاركتكم في هذا الاستبيان طوعيّ ولكم كل الحرية في الانسحاب من هذه الدراسة في أي وقت ترغبون بذلك. ولقد تمت الموافقة على هذه الدراسة من قبل لجنة الأخلاقيات ورقمها (00013692). وستكون جميع البيانات مجهولة المصدر دون اية اشارة لهويتك الشخصية.

هل توافق على تعبئة الاستبيان؟ □ نعم □ لا

1. **البيانات الاجتماعية**

|  | 1. بلد |
| --- | --- |
| □ مدينة □ ريف | 1. مكان السكن |
| --- | 1. العمر |
| □ ذكر □ انثى | 1. النوع |
| □ اقل من الشهادة الثانوية  □ شهادة ثانوية  □ شهادة جامعية او دراسات عليا | 1. اعلى شهادة تعليمية حصلت عليها |
| □ نعم □ لا | 1. هل تعاني من أي امراض مزمنة؟ |
| □ نعم □ لا | 1. هل تعاني من أي صعوبات تمنعك عن اداء الانشطة المعتادة نتيجة مشكلة صحية (مثال: صعوبة في المشي/صعود السلالم/الرؤية/السمع)؟ |
| □ صعب جداً □ صعب  □ محايد □ سهل  □ سهل جداً □ لا أستخدم الإنترنت | 1. ما مدى سهولة أو صعوبة استخدامك للإنترنت؟ |
| □ نعم □ لا | 1. هل كانت لديك تجربة سابقة مع خدمات الطب الإلكتروني؟ |
| □ تقييم التاريخ المرضي □ الفحص والمعاينة عن بُعد  □ تشخيص المرض □ متابعة عن بُعد  □ استشارة عن بُعد □ لا يوجد تجربة سابقة | 1. إذا كان جوابك بنعم، فما كان نوع الخدمات؟ (يمكنك اختيار أكثر من إجابة واحدة) |
| □ نعم □ لا | 1. هل تعتقد أنك ستستخدم إحدى خدمات الطب الإلكتروني في المستقبل إذا أتيحت لك الفرصة؟ |

1. **اعتقادك بخصوص استخدام خدمات** **الطب الإلكتروني**

للإجابة على القسم التالي اختر مما يلي: غير موافق اطلاقاَ - غير موافق - محايد - موافق- موافق بشدة

|  | غير موافق اطلاقاَ  1 | غير موافق  2 | محايد  3 | موافق  4 | موافق بشدة  5 |
| --- | --- | --- | --- | --- | --- |
| **مميزات استخدام** **الطب الإلكتروني:** | | | | | |
| 1. اعتقد أن خدمات الطب الإلكتروني ستُفيد في متابعة حالتي الصحية. |  |  |  |  |  |
| 1. اعتقد أن خدمات الطب الإلكتروني ستوفر الوقت |  |  |  |  |  |
| 1. اعتقد أن خدمات الطب الإلكتروني ستوفر المال |  |  |  |  |  |
| 1. اعتقد أن خدمات الطب الإلكتروني ستتيح لي أن أتلقى العلاج بشكل فعال |  |  |  |  |  |
| 1. اعتقد أن خدمات الطب الإلكتروني ستزيد من مستوى الراحة اثناء الوصول إلى خدمات الرعاية الصحية |  |  |  |  |  |
| 1. بشكل عام، أعتقد أن خدمات الطب الإلكتروني مفيدة |  |  |  |  |  |
| **مدى سهولة استخدام خدمات الطب الإلكتروني:** | | | | | |
| 1. اعتقد أن تعلم استخدام الطب الإلكتروني سيكون سهلاً. |  |  |  |  |  |
| 1. اعتقد أن استخدام نظام الطب الإلكتروني سيكون بسيطاً. |  |  |  |  |  |
| 1. اعتقد أن نظام الطب الإلكتروني سيكون واضح ومفهوم بالنسبة لي. |  |  |  |  |  |
| 1. بشكل عام، اعتقد أن استخدام خدمات الطب الإلكتروني سيكون مريحاً. |  |  |  |  |  |
| **تأثير الآخرين على موقفك تجاه استخدام خدمات الطب الإلكتروني:** | | | | | |
| 1. استخدام الأصدقاء والأقارب (الأشخاص المهمين بالنسبة لي) لخدمات الطب الإلكتروني سيؤثر على ما إذا كنت سأستخدمها. |  |  |  |  |  |
| 1. توصية الأصدقاء والأقارب (الأشخاص الذين لهم تأثير قوي عليّ) باستخدام خدمات الطب الإلكتروني ستؤثر على ما إذا كنت سأستخدمها. |  |  |  |  |  |
| 1. نصيحة الطاقم الطبي مثل الأطباء والممرضين لي باستخدام خدمات الطب الإلكتروني ستؤثر على ما إذا كنت سأستخدمها. |  |  |  |  |  |
| **الوسائل المساعدة لاستخدام خدمات الطب الإلكتروني:** | | | | | |
| 1. لدي الموارد اللازمة لاستخدام خدمات الطب الإلكتروني (مثال: انترنت). |  |  |  |  |  |
| 1. لدي المعرفة اللازمة لاستخدام خدمات الطب الإلكتروني (مثل كيفية العثور على منصة او تطبيق للطب الإلكتروني). |  |  |  |  |  |
| 1. أعتقد أن التعليمات المتعلقة باستخدام الطب الإلكتروني ستكون متاحة لي. |  |  |  |  |  |
| 1. أعتقد أن أشخاصاً محددين سيكونون متاحين لتقديم المساعدة عندما أواجه صعوبات في استخدام خدمات الطب الإلكتروني (مثل مركز الاتصال/ خدمة عملاء). |  |  |  |  |  |
| **القدرة على استخدام نظام الطب الإلكتروني:** | | | | | |
| 1. يمكنني استخدام خدمات الطب الإلكتروني بفضل تجربتي السابقة في تقنيات مشابهة. |  |  |  |  |  |
| 1. يمكنني استخدام خدمات الطب الإلكتروني بعد ملاحظة شخص آخر يستخدمها قبل تجربتها بنفسي. |  |  |  |  |  |
| 1. يمكنني استخدام خدمات الطب الإلكتروني بشكل مستقل، حتى في غياب الإرشادات من الآخرين. |  |  |  |  |  |
| 1. يمكنني تشغيل خدمات الطب الإلكتروني بالاعتماد على الكتيبات فقط كمرجع، حتى إذا لم يكن هناك أحد حولي. |  |  |  |  |  |
| **الشعور بالأمان** | | | | | |
| 1. سأشعر بالأمان عند إرسال معلومات صحية شخصية باستخدام الإنترنت وأجهزة الكمبيوتر. |  |  |  |  |  |
| 1. يوفر الإنترنت وسيلة آمنة لإرسال المعلومات الشخصية. |  |  |  |  |  |
| 1. سأشعر بالأمان التام عند تقديم معلومات شخصية عن نفسي عبر الإنترنت. |  |  |  |  |  |
| 1. بشكل عام، يعتبر استخدام أجهزة الكمبيوتر والاتصال بالإنترنت وسيلة آمنة لنقل المعلومات الصحية الشخصية. |  |  |  |  |  |
| **مساوئ الطب الإلكتروني:** | | | | | |
| 1. تكلفة الوصول إلى الإنترنت تعتبر عائقاً أمام استخدام خدمات الطب الإلكتروني. |  |  |  |  |  |
| 1. صعوبة استخدام التكنولوجيا تعتبر عائقاً أمام استخدام خدمات الطب الإلكتروني. |  |  |  |  |  |
| 1. استخدام خدمة الطب الإلكتروني تؤدى إلى عدم القدرة على إجراء فحص سريري. |  |  |  |  |  |
| 1. استخدام خدمة الطب الإلكتروني يمكن أن تنتهك خصوصية المريض. |  |  |  |  |  |
| 1. استخدام خدمة الطب الإلكتروني يمكن أن تزيد من الأخطاء الطبية. |  |  |  |  |  |
| ***رأيك عن الطب الإلكتروني بشكل عام:*** | | | | | |
| 1. أحب فكرة الطب الإلكتروني. |  |  |  |  |  |
| 1. سأوصي الآخرين باستخدام خدمات الطب الإلكتروني. |  |  |  |  |  |
| 1. لدي نية قوية لاستخدام/ الاستمرار في استخدام خدمات الطب الإلكتروني. |  |  |  |  |  |
| 1. إذا أتيحت لي الفرصة للوصول إلى خدمات الطب الإلكتروني، فإنني أنوي استخدامها. |  |  |  |  |  |

1. **اعتقادك وشعورك تجاه التكنولوجيا بشكل عام**

للإجابة على القسم التالي اختر مما يلي: غير موافق اطلاقاَ - غير موافق - محايد - موافق- موافق بشدة

| موافق بشدة  5 | موافق  4 | محايد  3 | غير موافق  2 | غير موافق اطلاقاَ  1 |  |
| --- | --- | --- | --- | --- | --- |
|  |  |  |  |  | 1. عموماَ، أنا لست من محبي التكنولوجيا. |
|  |  |  |  |  | 1. أشعر بالتردد في تعلم المميزات الجديدة الخاصة بالتكنولوجيا (مثال: تطبيق الكرتوني جديد- جهاز الكرتوني جديد). |
|  |  |  |  |  | 1. لا أشعر بالراحة عند استخدام التكنولوجيا. |
|  |  |  |  |  | 1. التكنولوجيا لا تُحسن جودة حياتي. |
|  |  |  |  |  | 1. أشعر بأنني خارج السيطرة/ غير متحكم عند استخدام التكنولوجيا. |
|  |  |  |  |  | 1. أشعر بعدم الارتياح عند استخدام التكنولوجيا. |
|  |  |  |  |  | 1. أرى أن التكنولوجيا تعقّد المهام البسيطة. |
|  |  |  |  |  | 1. يبدو أن مواكبة أحدث التقنيات أمر مستحيل. |
|  |  |  |  |  | 1. أنا غير كفء عند استخدام التكنولوجيا. |
|  |  |  |  |  | 1. استخدام التكنولوجيا يسبب لي التوتر/العصبية. |
|  |  |  |  |  | 1. استخدام التكنولوجيا يضايقني غالباَ. |

***شكراً جزيلاً على مشاركتكم القيمة***

**English version**

**Sociodemographic data**

| 1. Country |  |
| --- | --- |
| 1. Place of residence | Urban/ rural |
| 1. Age |  |
| 1. Gender | Male/ female |
| 1. Education | Less than secondary education  Secondary school graduate  University graduate or higher |
| 1. Chronic disease comorbidity | Yes/ no |
| 1. Disability that limit your daily activity | Yes/no |
| 1. How easy or difficult do you find it to use the Internet? | very difficult/ difficult/ neutral/ easy/ very easy/I don’t use the Internet |
| 1. Previous experience with telehealth services | Yes/no |
| 1. If yes, what kind of services? | Assessment/ examination/ diagnosis/ follow-up/ consultation/NA |
| 1. Do you think you would use one of the telehealth services in the future if you were offered the opportunity? (intention to use the telehealth services) | Yes/no |

**The Extended Unified Theory of Acceptance and Use of Technology (UTAUT) model**

Rating scale from 1 (strongly disagree) to 5 (strongly agree) then calculate the mean ±SD

| ***Performance expectancy:*** the degree to which an individual believes that using telehealth will help him or her increase their health performance/quality |
| --- |
| 1. Telehealth would be helpful in monitoring my health |
| 1. Telehealth can save my time |
| 1. Telehealth can save money |
| 1. Telehealth enables me to be effectively treated. |
| 1. Telehealth will enhance the level of convenience in accessing medical care services |
| 1. Overall, I think telehealth services are useful |
| ***Effort expectancy:*** the degree of ease associated with use of telehealth system |
| 1. I find that telehealth would be easy to learn |
| 1. I find that using telehealth would be simple |
| 1. I find that telehealth would be easily understandable and clear for me |
| 1. Overall, I find that using telehealth would be convenient |
| ***Social influence:*** |
| 1. The use of telehealth services by friends and relatives around me or by those who are important to me will influence whether I use it. |
| 1. The recommendation of friends and relatives around me or those who have a strong influence on me to use telehealth will influence whether I use it. |
| 1. Medical personnel such as doctors and nurses advise me to use telehealth will influence whether I use it. |
| ***Facilitating condition:*** reflect technical and organizational support |
| 1. I have the resources (e.g., network) necessary to use telehealth |
| 1. I have the knowledge necessary to use telehealth (e.g., how to find a telehealth platform). |
| 1. I believe instructions concerning use of the system will be available to me |
| 1. I believe specific persons (or a group) will be available for assistance when I have difficulties using telehealth (e.g., call center) |
| **Self-efficacy :** a person's belief in their ability to complete a task or achieve a goal |
| 1. I could use telehealth with previous experience in similar technologies |
| 1. I could employ telehealth after observing someone else using it before trying it myself |
| 1. I could use telehealth independently, even in the absence of guidance from others |
| 1. I could operate telehealth solely with manuals as references, even if there were no one around |
| **Perceived Security**: the degree to which using information technology enables the administration of personal health information |
| 1. I would feel secure sending personal health information using the Internet and computers |
| 1. The Internet offers a secure means through which to send sensitive personal information |
| 1. I would feel totally safe providing sensitive personal information about myself over the Internet |
| 1. Overall, using computers and an Internet connection is a safe way to transmit sensitive personal health information |
| ***Perceived barriers*** |
| 1. Cost and access to the Internet are obstacles against using telehealth services |
| 1. Difficulty in using Technology is an obstacle against using telehealth services |
| 1. Providing telehealth service leads to inability to have a physical check-up |
| 1. Providing a telehealth service can violate patient privacy |
| 1. Providing a telehealth service can increase medical errors |
| ***Intention to use*** |
| 1. I like the idea of telehealth |
| 1. I will recommend others to use the online learning |
| 1. I have high intention to use/ continue using telehealth services |
| 1. If provided with the opportunity to access telehealth services, I would intend to use it |

**The** **Abbreviated Technology Anxiety Scale**

Please respond to each prompt with the appropriate level of agreement per your personal feelings about yourself and technology. Reflective and open responses are encouraged and appreciated.

| Questions | Strongly agree | Agree | Neutral | Disagree | Strongly disagree |
| --- | --- | --- | --- | --- | --- |
| 1. I do not like technology in general. |  |  |  |  |  |
| 1. I am reluctant to learn new features of technology such as new application or new electronic device. |  |  |  |  |  |
| 1. I am uncomfortable using technology. |  |  |  |  |  |
| 1. Technology does not improve my quality of life. |  |  |  |  |  |
| 1. I feel out of control when using technology. |  |  |  |  |  |
| 1. I feel uneasy using technology. |  |  |  |  |  |
| 1. I feel technology complicates simple tasks. |  |  |  |  |  |
| 1. Keeping up with the newest technology is impossible. |  |  |  |  |  |
| 1. I am inefficient with technology. |  |  |  |  |  |
| 1. Using technology makes me nervous. |  |  |  |  |  |
| 1. I am often annoyed when using technology. |  |  |  |  |  |
